# Resting‐State Functional Interactions Between the Action Observation Network and the Mentalizing System

**DOI:** 10.1111/ejn.70082

**Published:** 2025-03-20

**Authors:** Luciano Simone, Enrica Pierotti, Eleonora Satta, Cristina Becchio, Luca Turella

**Affiliations:** ^1^ Department of Medicine and Surgery University of Parma Via Volturno 39 Parma Italy; ^2^ Center for Mind/Brain Sciences (CIMeC) University of Trento Rovereto (TN) Italy; ^3^ Laboratory for Autism and Neurodevelopmental Disorders, Center for Neuroscience and Cognitive Systems @UniTn, Istituto Italiano di Tecnologia Rovereto Italy; ^4^ Department of Neurology University Medical Center Hamburg‐Eppendorf Hamburg Germany

**Keywords:** action observation network, fMRI, functional connectivity, mentalizing system, resting state, social cognition

## Abstract

Human social functioning is thought to rely on the action observation network (AON) and the mentalizing system (MS). It is debated whether AON and MS are functionally separate or if they interact. To this end, we combined resting‐state connectivity with task‐based fMRI to characterize the functional connectome within and between these systems. In detail, we computed resting‐state connectivity within and between the AON and MS using single subject‐defined regions of interest (ROIs).

Our results showed a positive coupling between ROIs within each system and negative coupling between the two systems, supporting the existence of two independent networks at rest. Still, two regions (pSTS, aIFG) showed hybrid coupling, connecting with regions of both systems, suggesting that they might mediate cross‐network communication.

This characterization of the interplay between MS and AON in the healthy brain might provide the starting point to further investigate aberrant “connectivity” fingerprints associated with neuropsychiatric disorders characterized by impairments in social cognition.

## Graphical abstract

Our results describe the action observation network (AON) and the mentalizing system (MS) as two segregated networks, with two main sites of interaction—pSTS and aIFG—which might act as integration hubs between the two systems.

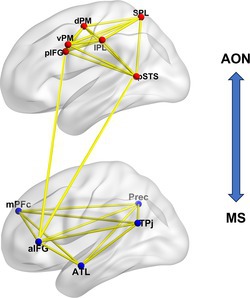

**Abbreviations**
AON: action observation network
aIFG: anterior inferior frontal gyrus
ATL: anterior temporal lobe
cs: central sulcus
dPM: dorsal premotor cortex
IPL: inferior parietal lobe
ips: inferior parietal sulcus
mPFc: medial prefrontal cortex
MS: mentalizing system
pIFG: posterior inferior frontal gyrus
Prec: precuneus
pSTS: posterior superior temporal sulcus
rs‐: resting‐state
SPL: superior parietal lobe
sts: superior temporal sulcus.
TPJ: temporo‐parietal junction
vPM: ventral premotor cortex

## 1 Introduction

The human capacity to infer the intentions underlying others' actions is a fundamental capability for successful social living. Two functional networks have been associated with processing action‐related social information: the action observation network (AON) and the mentalizing system (MS). Recent proposals suggest that most of our daily social functioning relies on the interaction between these two networks rather than on their independent functioning (Thioux, Gazzola, and Keysers 2008; Uddin et al. 2007; Van Overwalle and Baetens 2009). However, little is known about the candidate regions that could promote such interaction.

Previous research mainly investigated task‐based interactions between these two networks (Arioli et al. 2018; Becchio et al. 2012; Centelles et al. 2011; Ciaramidaro et al. 2014; de Lange et al. 2008), but it is still not clear whether the AON and MS interact even if participants do not perform any explicit task. Given their complementary nature, combining resting‐state and task‐based fMRI can provide a more comprehensive understanding of brain function by integrating insights from intrinsic connectivity and task‐induced activations (Cole, Smith, and Beckmann 2010; Hermundstad et al. 2013). For this reason, we aimed at characterizing whether and possibly how these functional networks interact by adopting a data‐driven approach, exploiting resting‐state (rs) fMRI connectivity analysis between brain regions identified with a standardized localizer within each network.

The AON is consistently reported to be active during action observation (Rizzolatti and Craighero 2004) and has been linked with the processing of various dimensions of observed actions, including kinematics, goal, and intention (see Kemmerer 2021, for a comprehensive review). The AON is readily engaged whenever a participant observes action stimuli across a variety of tasks (Caspers et al. 2010) and comprises a broad network of regions (Caspers et al. 2010; Keysers and Gazzola 2009; Turella et al. 2013), including the ventral premotor cortex (vPM), the dorsal premotor cortex (dPM), the posterior inferior frontal gyrus (pIFG), the superior parietal lobe (SPL), the inferior parietal lobe (IPL), and the posterior superior temporal sulcus (pSTS).

The MS is involved in actively inferring the mental states of others (Amodio and Frith 2006; Heleven and Van Overwalle 2018; Turella et al. 2013; Van Overwalle 2009; Van Overwalle and Baetens 2009). This network has been pinpointed in studies employing explicit social cognition tasks, such as attribution of intentions, emotions, and beliefs to other people (Amodio and Frith 2006; Frith and Frith 2006; Schurz et al. 2014). The brain regions consistently associated with this network include the medial prefrontal cortex (mPFc), the temporo‐parietal junction (TPJ), the precuneus (Prec), and anterior temporal lobe (ATL). In addition, a recent meta‐analysis (Schurz et al. 2014) also identified the most rostral part of the inferior frontal gyrus (aIFG) as part of this system (although some researchers regard this area as part of the “extended” AON, see Molnar‐Szakacs et al. 2005).

The abnormal engagement of one or both these networks have been associated with conditions characterized by impaired social functioning, such as autism spectrum and schizophrenia (Arioli et al. 2021; Weng et al. 2022). Moreover, large‐scale modifications of brain connectivity have been consistently described in these two conditions, i.e., autism (Müller and Fishman 2018) and in schizophrenia (Dong et al. 2018; Li et al. 2019). Network neuroscience provide a fruitful approach to explore psychiatric and neurodevelopmental disease, as brain network dysfunction is a potential hallmark feature characterizing these conditions (van den Heuvel and Sporns 2019; Bassett, Xia, and Satterthwaite 2018). Following this line of thinking, understanding the typical interactions within and between the two systems might be also helpful to further understand neuropsychiatric disorders characterized by specific impairments in social cognition. Indeed, although a large body of neuroimaging studies investigated the roles of individual regions of the AON and the MS in social cognition (Van Overwalle and Baetens 2009), little is known about the potential functional interplay within these two networks in healthy individuals. Some scholars hypothesize that the AON and the MS are largely segregated (Saxe 2005), whereas others advocate for their functional integration (Thioux, Gazzola, and Keysers 2008; Uddin et al. 2007; Van Overwalle and Baetens 2009), emphasizing that integration of information obtained from multiple domains—key for social inference tasks, such as intention attribution—requires communication paths between the two networks.

One way to address this ongoing debate is by evaluating the interactions between brain regions through functional connectivity analyses of fMRI data acquired while subjects perform no explicit task, i.e., rs connectivity (Buckner, Krienen, and Yeo 2013). In this type of connectivity analysis, the functional coupling between brain regions is described by computing pairwise correlations of their spontaneous low‐frequency BOLD signals at rest which is a measure of how the activity of these regions covaries in time. Despite some limitations (Howells et al. 2020), this approach has significantly advanced our comprehension of functional brain organization (Behrens and Sporns 2012). Here, we extend this approach to testing the balance between functional independence and interplay across AON and MS brain regions.

Our study explored the functional connectivity patterns within and between the regions of interest (ROIs) of the AON (vPM, dPM, pIFG, SPL, IPL, pSTS) and of the MS (TPJ, Prec, mPFC, ATL, aIFG). As it is not possible to identify all these ROIs purely on anatomical landmarks, we took advantage of single‐subject activation maps obtained through a standardized fMRI localizer (“Why/How localizer”, see Spunt and Adolphs 2014). This task‐based localizer allowed to identify the subject‐specific position of the ROIs within the AON and MS. Through this approach, we computed resting‐state connectivity tailoring the analysis on the specific functional characteristics of these networks for each participant.

The results of our rs connectivity analysis identify two largely segregated network, with two main sites of interaction ‐ pSTS and aIFG—which might act as integration hubs between the two systems. These findings provide the first description of the possible interactions between these two systems using subject‐specific connectivity analysis at “rest.”

## 2 Methods

### 2.1 Participants

The study included 17 subjects (8 females) with normal or corrected‐to‐normal vision. All participants were right‐handed with an average age of 25.94 years (range from 20 to 36 years). No participant reported a history of neurological disease or was taking psycho‐ or vasoactive medication.

The study was approved by the Ethics Committee of the University of Trento (Protocol number: 2018‐015) for research involving human participants. All participants were informed about the experimental procedures and provided written informed consent before experimentation.

### 2.2 Why/How Localizer

We adopted the Why/How localizer (Spunt and Adolphs 2014) to functionally define bilateral subject‐specific regions of the AON and the MS. In brief, participants were asked to answer questions about human behaviors presented in a set of pictures showing either intentional actions or emotional expressions. This paradigm embeds a 2 × 2 factorial design, with factors: stimulus category (faces, hands) and task (Why, How). The first factor manipulated the presented stimuli which represented either familiar facial expressions or common actions performed with the hand.

The second factor manipulated the type of task to be performed: In the Why task, participants had to answer by identifying the motive/intention (“why”) behind the observed behavior, while in the How task, participants had to answer questions about the means of “how” the observed behavior was performed (for further details, see Spunt and Adolphs 2014).

During the task, participants had to respond using an MR‐compatible response box depending on the requirements of the two tasks. The How task required to answer questions on the means of the observed interaction, e.g., “Is the person using both hands?”, whereas the Why task required to answer questions on the intentions behind the observed interaction, e.g., “Is the person helping someone?”. For a complete list of the questions, please refer to the original article (Spunt and Adolphs 2014).

The Why/How localizer started with 10 s in which participants had to fixate a central fixation cross (baseline) and ended with 30 s of similar “baseline” stimulation. The Why/How localizer comprises 16 trials (total duration 17 s each). Each trial began with a question (duration 1.5 s) providing the indication to the participant as to which task should be performed (either “How?” or “Why?”).

In each trial, eight pictures (duration 1.5 s each) were presented to participants in blocks. Between each picture, a reminder of the question for that block was presented (duration 0.5 s). The pictures were selected to represent one of the four possible combinations of stimulus category and task (How/Hand, How/Face, Why/Hand, Why/Face) within each trial. After each trial, a central fixation cross (duration 2 s) was presented as for the initial and final baseline stimulation. In total, there were four trials for each of the four experimental conditions (16 trials total). We adopted the original version of the localizer task downloaded from the official repository (https://www.bobspunt.com/whyhowlocalizer/) and modified the duration and interval between images, so that the duration of each block was fixed, instead of variable as in the original version of the localizer.

Participants performed well in the task (How task, 94.67%; Why task, 92.78%) and in line with the performance of the original work (Spunt and Adolphs 2014). Participants practiced the task outside the scanner with the “practice” version of the task (adopting different stimuli) which is included in the Why/How localizer.

Visual stimuli were presented on a liquid crystal monitor (Nordic NeuroLab, Norway; frame rate 60 Hz; screen resolution 1920 × 1080 pixels). Participants lay horizontally in the scanner and viewed the screen (31.2 x 17.5° of visual angle) via a rectangular mirror, positioned on the head coil. Button presses were collected via MR‐compatible response buttons (Nordic Neurolab Box, Nordic Neurolab, Norway). Stimulus presentation, response collection and synchronization with the scanner were controlled using MATLAB 2017 (MathWorks, Natick, MA, United States) and Psychtoolbox‐3 for Windows (Brainard 1997).

### 2.3 Experimental Session and MR Data Acquisition

Each participant completed one experimental session, comprising a structural anatomical scan (T1‐weighted image), two “Why/How localizer” scans and one resting‐state scan (rs‐fMRI).

In the two functional task‐based runs, participants performed the Why/How localizer task (see previous section). During rs‐fMRI scans, participants were not assigned any specific task. They were instructed to keep their eyes fixating on a fixation cross and stay awake during scanning.

Functional localizer and resting‐state data were collected using a 3 T Siemens MAGNETOM Prisma scanner equipped with a 64‐channel head‐coil. Functional data were acquired using a multiband EPI sequence (multi‐band acceleration factor 5, TR = 1 s, TE 28 msec, flip angle = 59°, matrix size = 100 × 100, in‐slice resolution 2 mm x 2 mm, 65 slices with 2 mm slice thickness with slice gap of 10%). We acquired 344 volumes for each functional localizer run and 614 volumes for the rs‐fMRI run. Functional localizer and rs‐fMRI runs were acquired with axial slices slightly tilted to be approximately parallel to the calcarine sulcus in order to optimize brain coverage. For each participant, we acquired a T1‐weighted anatomical scan (MPRAGE; TR 2140 ms; voxel resolution 1 mm x 1 mm x 1 mm; TE 2.9 ms; FA 12°; FOV 288 mm^3^; 208 slices).

### 2.4 MR Data Pre‐Processing

Data were preprocessed and analyzed with the Statistical Parameter Mapping package, SPM12 (Wellcome Department of Cognitive Neurology, London, United Kingdom), implemented in Matlab 9.1 (The MathWorks). For both the localizer and the RS data, functional images were corrected for slice time and motion, coregistered with the high‐resolution anatomic scan, normalized into the MNI space, resampled at 2x2x2 mm and smoothed with a Gaussian kernel of 8 mm full‐width half‐maximum. After these common pre‐processing steps, the rs‐fMRI timecourse were band‐pass filtered between 0.008 and 0.09 Hz. In addition, motion was regressed out to diminish its impact.

### 2.5 Why/how Localizer: Univariate Analysis

We performed univariate analyses, both at subject and group‐level, to functionally identify the ROIs using the Why/How localizer task (Spunt and Adolphs 2014). We analyzed both localizer runs and adopted a general linear model to identify the brain regions activated during Why/How localizer task execution. To evaluate the blood‐oxygen‐level‐dependent (BOLD) response associated with the performance of the Why/How localizer, we created regressors for each combination of stimulus category (faces, hands) and task (Why, How). These 4 conditions of interest were modelled as a block considering the duration of all the picture repetitions in the trial (duration 17 s). In addition, motion parameters (three for rotation, three for translation) were included in the model as regressors of no interest.

Contrast of interest from the first level analysis were then entered in a second level analyses to assess group‐level effects with a one‐sample t‐test performed with a non‐parametric approach (SnPM13, http://www.fil.ion.ucl.ac.uk/spm/snpm). The second‐level results were corrected for multiple comparisons using a false discovery rate (FDR; *p* value < 0.005).

To identify MS, we adopted a task comparison (t‐contrast: Why task > How task, see Spunt and Adolphs 2014). The rationale was that in the Why task, participants had to attribute a mental state to the observed human actors (intention, emotional state) by engaging MS, whereas the How task did not include mental‐state attribution (i.e., participants had to focus on aspects of the body state/actions performed by the actors in the images).

The topography of our results was in line with the original results of the study by Spunt and Adolphs (Spunt and Adolphs 2014, see images of the original article at (https://www.bobspunt.com/whyhowlocalizer/). Moreover, the set of cortical regions identified by this contrast are functionally consistent with meta‐analytic definitions of the mentalizing network (Schurz et al. 2014).

The Why task > How task contrast showed significant activation within early visual areas, the precuneus, the superior, middle and inferior frontal gyrus, the cingulate cortex, middle temporal gyrus along its anteroposterior direction (height threshold, non‐parametric *p* value alpha = 0.0182, equivalent to *p* < 0.005 FDR‐corrected).

To identify AON, we considered activation elicited by all the stimuli irrespective of the task, as they depicted different types of human interactive behaviors (t‐contrast: Why + How task vs. baseline). We adopted the contrast Why + How task vs. baseline, as both tasks required to observe the same stimuli and it is known that the AON is activated by passive observation of the stimuli regardless of the specific task in which participants are engaged (“How” or “Why”). A different contrast, e.g. How task > Why task, would have highlighted regions within and/or outside the AON which are required to infer the means (“How”) by which an action is performed. Still, this choice might highlight regions modulated also by the motor response and not only associated with action observation.

The cortical regions activated by this contrast were consistent with meta‐analytic study of AON in humans (Caspers et al. 2010) and in line with many neuroimaging studies on this topic (Becchio et al. 2012; Koul et al. 2018; Turella et al. 2012, 2009).

This contrast showed significant activation within early visual regions, the posterior portion of superior temporal sulcus, inferior and superior parietal regions, left primary motor cortex, lateral and medial premotor cortex and the inferior frontal gyrus (height threshold, non‐parametric *p* value alpha = 0.0010 equivalent to *p* < 0.005 FDR‐corrected).

### 2.6 RS Analysis: Selection of ROIs

For each hemisphere, we identified five mentalizing‐related regions: mPFc, aIFG, ATL, Prec, and TPJ. In addition, we bilaterally identified six action‐observation‐related regions: pIFG, vPM, dPM, IPL, SPL, and pSTS.

The selection of both mentalizing‐related and action‐observation regions was based on previous meta‐analysis studies (Caspers et al. 2010; Schurz et al. 2014). We identified functional bilateral ROIs from Why > How and Why + How contrasts, first at the group level and then at single level through the following procedures.

At the group level, we first calculate a mentalizing and action‐observation mask based on second‐level results analyses. We employed the contrast Why > How for isolating mentalizing regions and Why + How to identify action observation related regions. Then, we extracted MNI coordinates of the peak activation of mentalizing and action‐observation regions within the probabilistic maps of cytoarchitectonic boundaries provided by Juelich Histological Atlas (SPM Anatomy toolbox, Eickhoff et al. 2005). More specifically, with this atlas, we isolated three mentalizing regions (mPFc, aIFG, and Prec) and four action observation regions (pIFG, dPM, IPL, SPL). Notably, although aIFG has been also described as involved in action observation, we decided to include it among the mentalizing regions based on a metanalysis on MS (Schurz et al. 2014) and to ascertain possible differences in functional connectivity with the adjacent pIFG, which is most commonly associate with task involving observation of action. The position of the ROIs not defined in the Juelich Histological Atlas (ATL, TPJ, vPM, pSTS) were identified selecting the closest local maxima of mentalizing and action observation masks to the coordinates of mentalizing and action‐observation regions identified through meta‐analyses (Caspers et al. 2010; Schurz et al. 2014). Finally, TPJ coordinates were defined by combining the peak activation of mentalizing mask with right TPJ tractrography‐based parcellation ROI provided by Mars et al. (2012). We flipped right TPJ tractrography‐based ROI to identify TPJ peak activation in the left hemisphere.

Because of weaker activity, the group‐level peak of activations of right inferior and superior parietal regions from Why + How contrast was identified by slightly lowering the threshold. For this procedure, single‐subject maps were thresholded at *p* < 0.001 uncorrected (lowest T‐value 3.3). For two subjects, it was necessary to lower this threshold down to *p* < 0.02 uncorrected (lowest T‐value 2).

The group‐level coordinates of mentalizing and action‐observation regions are reported in Table 1.

**TABLE 1:** Average position of the ROIs.

AON	Left			Right		
dPm	−26	−8	52	30	−8	51
vPm	−58	6	36	50	6	42
pIFG	−46	8	30	52	11	26
IPL	−56	−24	40	36	−46	46
SPL	−28	−54	60	28	−60	58
pSTS	−54	−54	8	52	−50	12

MS	Left			Right		
mPFc	−6	52	28	6	54	16
ATL	−62	−8	−12	52	−2	−20
Precuneus	−6	−56	36	3	−56	38
TPj	−60	−58	26	53	−56	23
aIFG	−54	30	8	48	26	22

This procedure allowed us to locate mentalizing and action‐observation regions estimated at the population level. Then, the group‐level coordinates of mentalizing and action observation regions were used to identify single‐subject‐level ROI location. Indeed, we localized the coordinates of mentalizing and action observation‐related ROIs by identifying the single‐subject‐level local maxima within a radius of 8 mm centered on group‐specific ROIs coordinates as obtained from the first procedure.

Subject‐specific peak coordinates were used as input (8 mm radius spheres) in subsequent ROI‐to‐ROI connectivity analyses at individual level respectively. Although we performed volume‐based analysis, to better represent the spatial relationship between ROI and sulci location, the position of the ROIs are represented on an inflated representation of the brain surface.

### 2.7 RS Analysis: ROI‐to‐ROI Functional Connectivity

To reveal the functional interplay within and between MS and AON, we computed pairwise correlations among the timecourses of the functionally identified subject‐specific ROIs. Specifically, we first studied the functional connectivity using rs‐fMRI data between the ROIs identified with the Why/How localizer. To this end, we performed second level ROI‐to‐ROI connectivity analyses using subject‐specific coordinates from the Why/How localizer task.

ROI‐to‐ROI correlational analyses were performed using the functional connectivity (CONN) toolbox (https://web.conn‐toolbox.org/, Whitfield‐Gabrieli and Nieto‐Castanon 2012), a MATLAB/SPM‐based cross‐platform open‐source software.

ROI‐to‐ROI analysis was performed by calculating the temporal correlation between the average blood‐oxygen‐level‐dependent (BOLD) signals of all pairs of ROIs selected using our first approach (see previous section). Fisher r to Z transformation was applied to all the correlations coefficients of the ROI pairs. For each subject, we built a group‐specific ROI‐to‐ROI connectivity matrix in which any element represents the Fisher‐transformed bivariate correlation coefficient between a pair of ROIs BOLD timeseries.

Moreover, the CONN toolbox provides the possibility to perform network‐based statistic (NBS, Zalesky, Fornito, and Bullmore 2010) to control for false positives at the family‐wise level which is particularly suitable when analyzing the entire functional connectome.

Single‐subject ROI‐to‐ROI connectivity matrices were then entered into a second‐level general linear model to obtain population‐level estimates allowing estimation of a group‐specific ROI‐to‐ROI correlation matrix.

Since in the ROI‐to‐ROI analysis, our hypothesis involved the connection between a large set of ROIs, we corrected to control for multiple comparisons across all possible ROIs connections by using a connection‐level threshold (FDR‐corrected *p* < 0.05).

As a control analysis, we performed the same connectivity analysis on a sample of 983 right‐handed participants coming from the Human Connectome Project (see supplementary materials). We extracted the same pairwise connectivity matrices from the ROIs identified in our localizer tasks (see Table 1 for the MNI coordinates of the peak). The resulting connectivity matrices were in line with the one extracted from our sample (see Figure S1).

### 2.8 RS Analysis: Hierarchical Clustering

To reveal the network organization of MS and AON, we also used a data‐driven procedure. The hierarchical clustering algorithm (Bar‐Joseph, Gifford, and Jaakkola 2001) implemented in CONN toolbox (Whitfield‐Gabrieli and Nieto‐Castanon 2012) computed a similarity matrix between all pairs of input elements to be clustered based on ROI‐to‐ROI functional similarity metrics. Differences between each pair of ROIs were calculated as a weighted average of differences in connectivity statistics (functional criteria).

Finally, we also applied the “clusterdata” function to explore our connectivity fingerprints, which is a MATLAB‐based algorithm that identifies a multilevel hierarchy of clusters by creating a cluster tree.

## 3 Results

### 3.1 Intra‐Hemispheric Functional Connectome of the MS and AON

**FIGURE 1:**
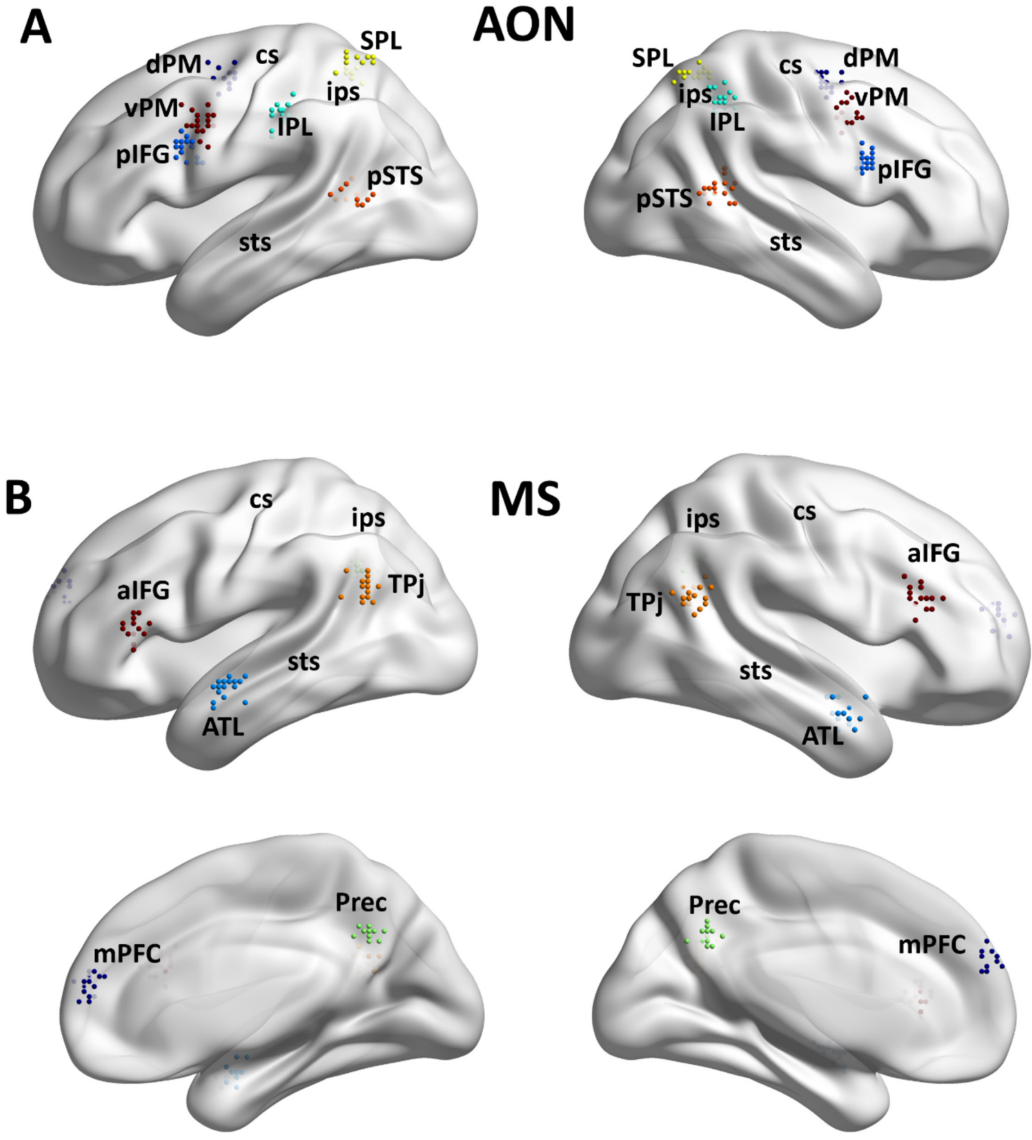
Position of the ROIs for the two networks. The positions of the subject‐specific ROIs of the AON (A) and the MS (B) are depicted on 3D brain images. The position of each ROI was determined by extracting the peak of activations coordinates obtained from the Why + How contrasts (A) and Why > How (B) of our standardized localizer (see Methods for details). Abbreviations: cs, central sulcus; ips, inferior parietal sulcus; sts, superior temporal sulcus.

Our study explored the functional connectome within and between specified regions of interest (ROIs) of the AON (vPM, dPM, pIFG, SPL, IPL, pSTS) and of the MS (TPJ, Prec, mPFc, ATL, aIFG). To provide an unbiased definition of the two networks, we relied on two meta‐analyses investigating the AON (Caspers et al. 2010) and the MS (Schurz et al. 2014). Given that not all specified ROIs are identifiable purely on anatomical landmarks, we combined single‐subject activation maps obtained through a standardized fMRI localizer (the “Why/How localizer,” see Spunt and Adolphs 2014) with coordinate‐based metanalysis (Caspers et al. 2010; Schurz et al. 2014) to functionally identify the subject‐specific position of the ROIs within AON and MS. Through this approach, we tailored our analysis on the specific functional characteristics of these networks for each participant (see Methods section for more details). The position of the ROIs of the MS and the AON for each participant are shown in Figure 1A,B (see also Table 1 for the group‐level MNI coordinates).

Next, we computed the pairwise functional connectivity between the five MS regions and the six AON regions in each hemisphere at the single‐subject level. The subject‐specific ROI‐to‐ROI connectivity matrices obtained by computing the pairwise functional coupling of all the functional ROIs were then tested at the group level.

**FIGURE 2:**
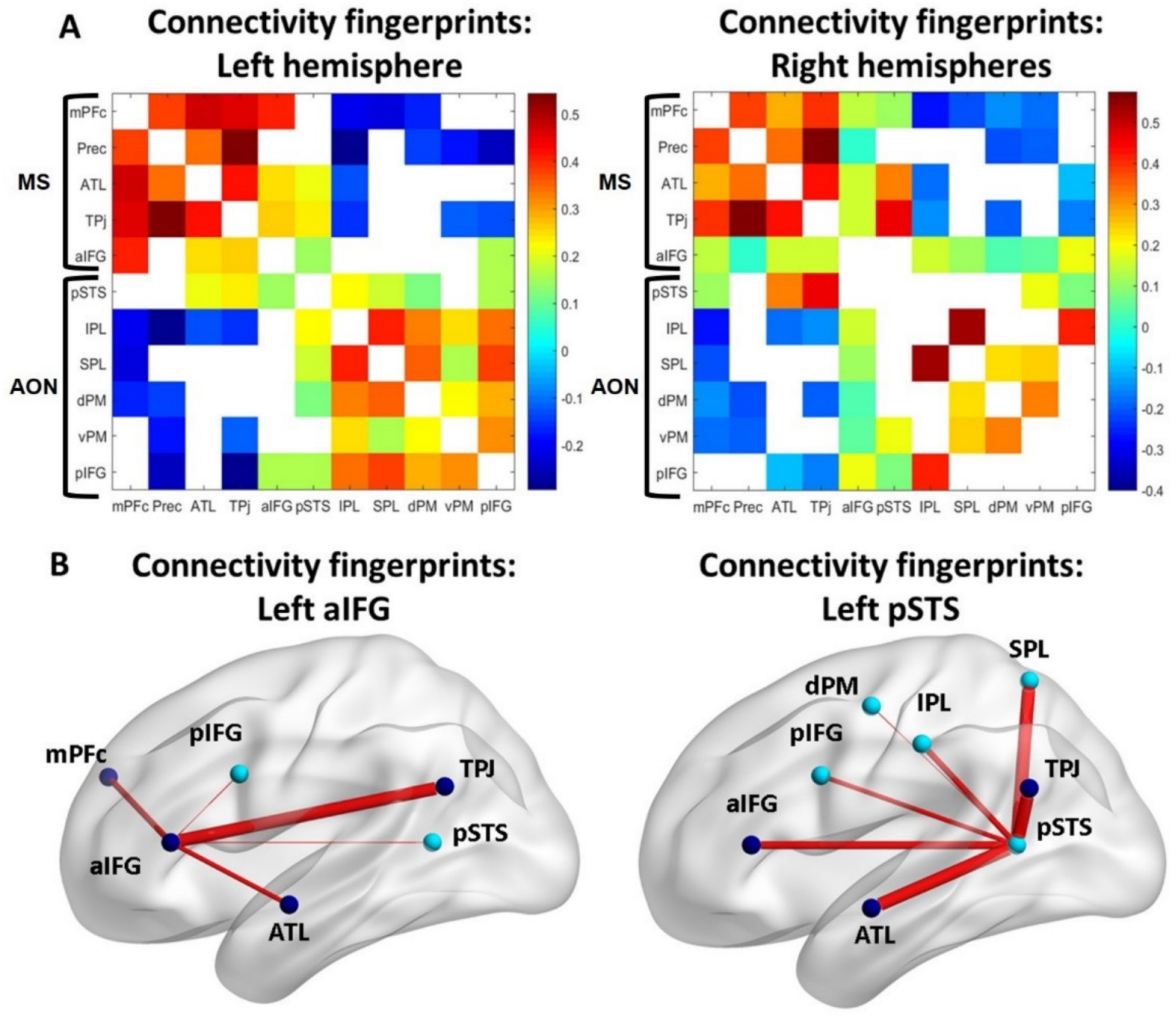
Intrahemispheric rs functional connectivity of AON and MS functional ROIs. (A) The correlation matrices for the ROIs of the MS and of the AON were considered separately for the left and right hemispheres. All significant correlations are reported in jet colormap whereas non‐significant correlations are shown in white (FDR‐corrected *p* value < 0.05). The diagonal (correlation of each ROI with itself) is represented in white. Color bar represents (Fisher z‐score) Pearson correlations. (B) As an example, we report a graphic representation of the positive correlations of aIFG and pSTS of the left hemisphere. In addition to the expected connectivity with AON regions (light blue spheres in (B)), pSTS was also functionally coupled with MS regions (dark blue spheres in (B)). The aIFG was positively correlated not only with MS regions—as expected—but also with AON ROIs.

Figure 2 shows the correlation matrices computed using the ROIs of the AON and of the MS of the left hemisphere and of the right hemisphere (Figure 2A). Significant correlations between ROIs are shown in jet colormap, whereas non‐significant correlations are shown in white. The diagonals (auto‐correlations) are not meaningful, because they represent the correlations of the timecourse of each ROI with itself (auto‐correlations), so they are shown in white.

By considering the right and left hemispheres separately, there were 38 and 36 significant ROI‐to‐ROI pairwise functional correlations respectively (out of a total of 55 per hemisphere, see Figure 2A). Overall, intra‐hemispheric functional connectivity of the AON and MS ROIs exhibited a highly similar connectivity pattern across the hemispheres, suggesting a consistent functional architecture.

Within each hemisphere, nearly all the mentalizing ROIs (mPFc, ATL, Prec, TPJ) correlated positively with the other MS functionally identified ROIs and were either uncorrelated or negatively correlated with AON ROIs (Caspers et al. 2010). This functional connectivity pattern was consistent across hemispheres (Figure 2A).

Similarly, across both hemispheres, nearly all AON ROIs (dPM, vPM, pIFG, SPL, IPL), identified by the Why/How localizer, were correlated positively with other AON ROIs and were either uncorrelated or negatively correlated MS ROIs.

Only two ROIs—the aIFG and pSTS—exhibited distinctive patterns of connectivity. Specifically, the aIFG, while generally positively correlated with other MS regions to which it belongs, also showed positive correlations with the parietal, temporal and frontal AON regions (Figure 2A). Similarly, the pSTS, although part of the AON and positively correlated with other AON ROIs, also exhibited positive correlations with the MS regions, including TPJ and ATL (Figure 2A). A spatial plot of the functional connectivity patterns of the left aIFG and left pSTS is shown in Figure 2B.

### 3.2 Inter‐Hemispheric Functional Connectome of MS and AON

**FIGURE 3:**
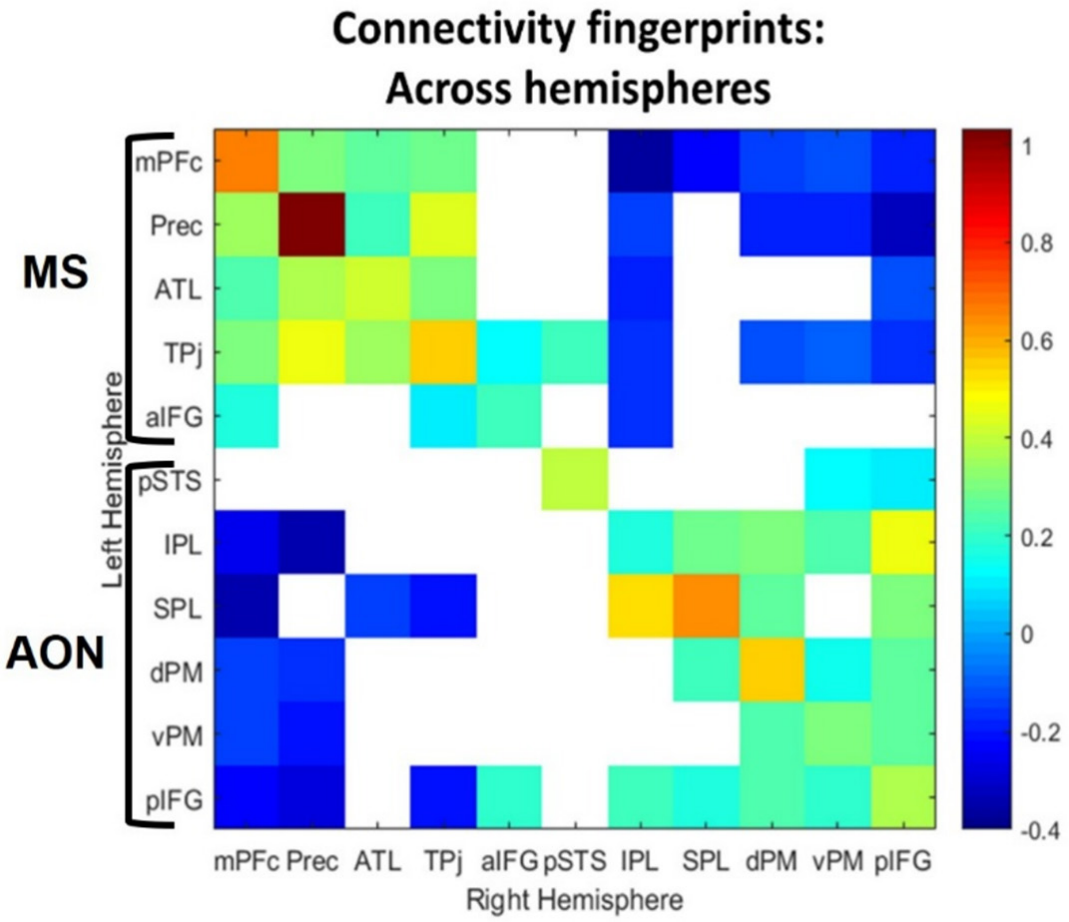
Interhemispheric connectome of MS and AON. The correlation matrix for the ROIs of the MS and of the AON was also computed across hemispheres. All significant correlations are reported in jet colormap whereas non‐significant correlations are shown in white (FDR‐corrected *p* value < 0.05). Color bar represents (Fisher z‐score) Pearson correlations.

We also explored inter‐hemispheric functional connections and computed group‐specific ROI‐to‐ROI analyses between regions located in different hemispheres. Out of 121 ROI‐to‐ROI pairwise functional correlations, 74 were statistically significant. Figure 3 shows the correlation matrix representing significant functional coupling of MS and AON ROIs of the left hemisphere to their homologous regions in the right hemisphere (significant connections: jet colormap, not significant: white).

The MS and AON ROIs showed a similar pattern of inter‐hemispheric functional connectivity. Indeed, MS and AON ROIs were positively correlated with contralateral regions belonging to the MS and AON, respectively, and were uncorrelated or negatively correlated with contralateral regions of the other functional network (Figure 3).

### 3.3 Hierarchical Clustering of the ROIs of the AON and MS

Our functional connectivity results confirmed that the ROIs clustered into two distinct functional systems: the MS (mPFc, Pre, ATL, TPJ) and the AON (vPM, dPM, pIFG, IPL, SPL). Only two regions, aIFG (included in the MS) and pSTS (included in the AON), showed “hybrid” connectivity patterns. These regions demonstrated functionally diverse connectivity fingerprints and were positively correlated with regions belonging to the other functional network (i.e., AON for aIFG, MS for pSTS).

To further validate these results, we employed a data‐driven methodology involving hierarchical clustering based on the similarity of ROI‐to‐ROI functional connectivity. This analysis allowed us to independently test the hierarchical organization of resting‐state connectivity of the AON and MS shown above.

**FIGURE 4:**
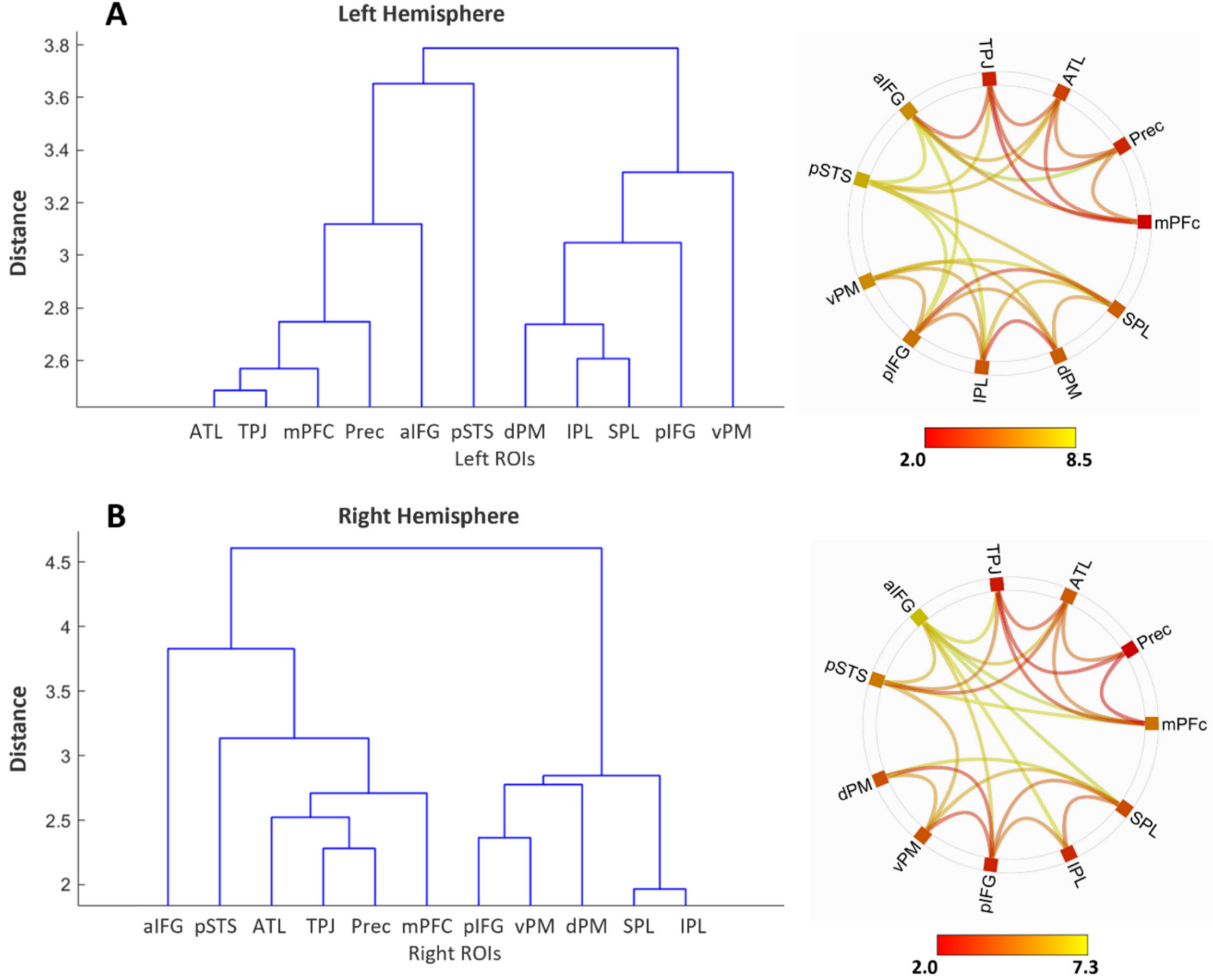
Hierarchical clustering. The right (A) and left (B) functional ROIs of MS and AON were clustered according to a data‐driven algorithm. Color bar represents *t* values.

Figure 4 shows the results of the hierarchical clustering analysis applied to the similarity between all pairs of ROI functional connectivity.

The clustering produced similar results in both left (Figure 4A) and right (Figure 4B) hemisphere. Indeed, the clustering algorithm grouped vPM, dPM, IPL, SPL, and pIFG in a specific cluster, while mPFc, ATL, and TPJ were grouped in a second functional network. Finally, the hierarchical clustering algorithm confirmed the heterogeneity of the functional connectivity of aIFG and pSTS; the left and right pSTS clustered with the mentalizing regions, whereas aIFG clustered with MS regions in the left hemisphere and with AON regions in the right hemisphere.

## 4 Discussion

Previous neuroimaging investigations have proposed that two distinct networks, the AON and the MS, contribute to social cognition (Thioux, Gazzola, and Keysers 2008; Uddin et al. 2007). However, the question of whether these systems function independently or interact with each other remained unresolved. Here, we exploited resting‐state fMRI connectivity to understand the functional relationship between the AON and the MS.

Previous investigations focused mainly on investigating possible task‐based connectivity interplay between these two networks (Arioli et al. 2018; Becchio et al. 2012; Centelles et al. 2011; Ciaramidaro et al. 2014; de Lange et al. 2008). Still, the structure of the functional connectivity during task performance has been suggested to be composed by an intrinsic network architecture—already present at rest—which is then shaped by task‐general and task‐specific connectivity modifications (Cole et al. 2014). The understanding of this “intrinsic” connectivity architecture between the AON and the MS was the main and novel aim of our research.

It has been proposed that to develop a comprehensive neurocognitive functional framework using resting‐state networks, it may be useful to combine task‐ and resting‐state fMRI by adapting task‐based methods to enhance the insights gained from studies of spontaneous activity patterns or RSNs (Cole, Smith, and Beckmann 2010). The strict relationship between task‐based and resting‐state fMRI, as described here for the first time in AON and MS, could open new avenues for studies in social perception.

By combining subject‐specific ROIs definition and resting‐state connectivity analyses, our work offers three novel insights into the functional interactions within and between these two networks.

Firstly, we show a strong positive coupling between ROIs within each system. This pattern of functional connectivity supports the idea that each system has a modular architecture.

Secondly, most of the ROIs within each system showed anticorrelated connectivity patterns with regions of the other system. This finding, confirmed by our data‐driven clustering approach, bolsters the idea that the two networks are at least partly segregated.

Thirdly, two regions—the pSTS and the aIFG—also showed increased functional connectivity with regions of the other network supporting the idea that they act as key gateways for information transfer and integration between these two systems.

In the following sections, we will discuss the implications of our findings in light of recent advances in the field of social cognition.

### 4.1 Functional Interactions Within the AON and the MS

Our connectivity results confirmed the existence of two separate functional connectomes: one for the AON—comprising vPM, dPM, pIFG, SPL, IPL, and pSTS—and one for the MS—composed of mPFc, ATL, Prec, TPJ, and aIFG.

While numerous functional studies have proposed different, yet potentially complementary, roles of the MS and AON in social cognition, only few studies have explored their functional connectivity architecture at rest (Marchetti et al. 2015; Molinari et al. 2013). Our study is the first to provide evidence that rs functional connectivity can be adopted to segregate the AON and MS. This characterization of the functional interaction between these two systems is complementary to the data obtained with meta‐analyses showing consistent recruitment of AON regions during action observation (Caspers et al. 2010; Hardwick et al. 2018) and MS regions during theory‐of‐mind tasks (Molenberghs et al. 2016; Schurz et al. 2014).

Our findings gain further credibility from anatomical connections between regions within these networks, as described in human studies using white matter tractography. For instance, recent investigations have shown that the superior longitudinal fasciculus, a large‐association bundle composed of medial and lateral fibers, connects the frontal, parietal, and temporal components of AON (Wang et al. 2018). Particularly in the left hemisphere, the functional connectivity pattern between parietal, premotor and temporal regions of the AON overlaps with the one previously identified using tractography (Howells et al. 2020).

Regarding the MS, tractography‐based studies suggest that mentalizing functions are primarily supported by two tracts: the cingulum bundle, an association white‐matter pathway that connects medial nodes of TOM (mPFC‐Prec) and the frontal cortex with temporal lobe (mPFc‐ATL), and the arcuate fasciculus, which projects to lateral nodes of TOM, thus connecting frontal cortices with temporal regions (uncinate fasciculus, IFG‐ATL, inferior frontal occipital fasciculus, TPJ‐IFG, see Wang et al. 2018).

Interestingly, two similarly organized networks also emerge also from rs connectivity of macaque monkeys (Yokoyama et al. 2021), suggesting that not only the AON, but a precursor of the MS might be also present in other non‐human primates, challenging conventional beliefs.

### 4.2 Functional Interactions Between the two Systems

Previous fMRI studies adopting task‐based analyses have suggested the interplay between these the AON and the MS to be a key factor in social cognition, particularly, when inferring others' intentions (Arioli et al. 2018; Becchio et al. 2012; Centelles et al. 2011; Ciaramidaro et al. 2014; de Lange et al. 2008) and during online social‐interaction tasks (Schippers et al. 2010; Sperduti et al. 2014).

The proposal for an interplay between MS and AON has also been supported by task‐related effective connectivity studies (Tettamanti et al. 2017; van Ackeren, Smaragdi, and Rueschemeyer 2016) showing increased interaction between these two networks when participants were asked to infer others' intentions or beliefs. These studies indicate mPFc (van Ackeren, Smaragdi, and Rueschemeyer 2016) and left and right IFG (Tettamanti et al. 2017) are responsible for the coupling between AON and MS systems during task performance. Abnormal task‐related interactions between the AON and the MS have been reported in autism (Cole, Barraclough, and Andrews 2019).

These investigations are of paramount importance as they described the interactions between the two systems through task‐based paradigms. Still, these paradigms are strongly influenced by task requirements and characteristics, making it challenging to generalize their findings. Indeed, a recent review (Schurz, Maliske, and Kanske 2020) showed that meta‐analytical maps of specific mentalizing tasks (Schurz et al. 2014) comprise areas of different fMRI resting‐state networks (e.g., default mode network, Sensorimotor Network, see Yeo et al. 2011).

Our study provides complementary information by investigating the two networks at rest and by adopting specific ROIs identified using a localizer task instead of focusing on large‐scale networks. Exploiting rs connectivity, our approach aimed to establish task‐free relations that might represent the “basic” interactions between these two systems. Using this approach, we were able to reveal that even at rest the two systems communicate through two common nodes—the pSTS and the aIFG. In the following sections, we elaborate on the possible role of these two regions.

### 4.3 Functional Interactions Between the two Systems: Role of pSTS

The superior temporal sulcus (STS) comprises functionally distinct subregions, each specialized for a specific domain of social perception (Deen et al. 2015). Its posterior section (pSTS) encodes information about biological motion and actions performed with different effectors, such as eye, hand, face, and body (Allison, Puce, and McCarthy 2000; LaBar 2003; Pitcher et al. 2011). It has been proposed as a key hub of a third visual pathway—in addition to the ventral and dorsal streams—specialized in representing dynamic aspects of observed social stimuli (Pitcher and Ungerleider 2021). The pSTS is also part of a wider region—the lateral occipitotemporal cortex (LOTC, Lingnau and Downing 2015)—which has been consistently associated with the representation of action and object information and which is organized along different encoding gradients (Lingnau and Downing 2015; Wurm and Caramazza 2022).

Specific regions within the pSTS activated during action observation have traditionally been viewed as the entry node to the AON (Rizzolatti, Fogassi, and Gallese 2001; Rizzolatti and Craighero 2004), providing high‐order visual representation of observed actions to parietal regions (IPL), from which the information is then transferred to vPM.

With respect to MS, meta‐analytical analysis provides evidence for a concurrent recruitment of pSTS in task engaging both systems (Yang et al. 2015). Similarly, multifunctional overlap analysis shows a consistent overlap between responses to face perception and mental‐state understanding (Deen et al. 2015). Our results further support the notion that the pSTS serves not only as key region of the MS system but also as a communication hub between the MS and the AON.

The strong positive correlation with TPJ speaks to a direct exchange of information between these two regions, such that “social” information regarding the observed scene might be further processed within TPJ. TPJ has classically been considered a crucial hub of MS recruited in a wide range of mentalizing tasks, particularly when we are engaged in inferences of others' beliefs and thoughts (Heleven and Van Overwalle 2018; Saxe and Kanwisher 2003). In line with the idea of strong interactions between these two regions, a recent review (Patel, Sestieri, and Corbetta 2019) proposed that the pSTS‐TPJ might be part of a pathway underlying uniquely human social abilities. Indeed, the rearrangement and expansion of the pSTS‐TPJ complex in our species—compared to non‐human primates—would be a key hallmark of human social capabilities, providing the backbone for processing complex dynamic social interactions.

The strong positive correlations between pSTS and ATL/IFG point towards a possible pathway for information transfer between pSTS and frontal regions. Information might be transferred from pSTS not only to IPL and TPJ, but also to prefrontal cortex, i.e., IFG either directly or indirectly passing through ATL.

The existence of a flow of visual information from the temporal cortex to IFG has been proposed by Kilner (2011) in a model in which pMTG—an area within the LOTC—transfers information to IFG and from there to vPM. This pathway would act in parallel to the flow of information from temporal to parietal regions and then to premotor cortex assumed in the classical AON model (Rizzolatti and Craighero 2004). In Kilner's model, the two pathways are proposed to mediate complementary roles in action understanding. Building on our results, we propose that the flow of information from temporal cortex to IFG may also serve as pathway of communication between MS and AON, as information could be further exchanged among prefrontal regions, such as mPFc.

### 4.4 Functional Interactions Between the two Systems: The Role of aIFG

The other region showing an interplay with both systems was aIFG. In general, IFG has been associated with both AON (Rizzolatti and Craighero 2004) and MS (Schurz, Maliske, and Kanske 2020; Schurz et al. 2014). The current view describes IFG as organized along a rostro–caudal gradient implying a hierarchical organization of this region (Badre and D'Esposito 2009) with the more abstract features of the observed action implemented anteriorly and the more concrete features of the observed actions encoded posteriorly (de Vignemont and Haggard 2008).

On one hand, our data suggests that pIFG is part of the AON as it shows positive correlation with the other AON regions and no connectivity with MS, apart from strong connectivity with aIFG. On the other hand, aIFG shows strong connectivity with the MS regions and with regions of the AON (particularly in the right hemisphere). This supports the possibility that aIFG is a site for interactions between the two systems, but its role in mediating these interactions is still unclear.

Previous meta‐analyses by Schurz, Maliske, and Kanske (2020); Schurz et al. (2014) highlighted a specific recruitment of the IFG mainly in two specific social cognition tasks, i.e., social animations and mind‐in‐the‐eyes. Social animations tasks involve the observation of geometric shapes moving following specific patterns which look like individuals involved in social interactions. Mind‐in‐the‐eyes tasks require participants to infer the mental state of a person from eye region. As these tasks require the prediction of mental states or intentions from visual stimuli, it has been proposed that MS might be supported by AON. In this context, aIFG might provide information processed from AON to other MS regions or to enable a top‐down modulation from the MS on the processing of actions within the AON.

An example of this possible role of the IFG comes from an fMRI investigation adopting a real interaction paradigm (Cavallo et al. 2015). Either participants in the scanner had to exchange eye contact with a partner in the scanner room (via a mirror), or both participants were asked to avert their gazes away from each other. This study showed that the interaction between IFG and mPFc increases when subjects process direct gaze during a real interaction, thus suggesting the involvement of these areas in mediating the communicative intent represented visually by eye direction (Cavallo et al. 2015).

Following this possibility, recent studies support the idea that IFG is strongly reliant on its interactions with mPFc during mentalizing tasks. Indeed, mPFc demonstrated significantly increased connectivity with right pIFG/vPMC when motive was inferred from videos, as compared to text descriptions of actions (Spunt and Lieberman 2012). This finding is also in line with recent meta‐analysis, showing stronger recruitment of the IFG in theory‐of‐mind tasks which involved visual stimulation rather than textual stimuli (Molenberghs et al. 2016). Furthermore, a recent effective‐connectivity study showed that there might be a difference in specialization based on lateralization for IFG (Tettamanti et al. 2017). Mentalizing based on linguistic input or on non‐linguistic information influences the activity in MS areas (mPFc, TPJ, Prec) differently. The left IFG seems to be particularly relevant when linguistic information needs to be provided to the MS regions, whereas right IFG might act as an extralinguistic modality‐specific gateway.

## 5 Conclusions

Overall, our findings offer crucial insights to theories of social cognition and intention understanding by showing that (a) the MS and AON are characterized by specific patterns of connectivity, i.e., functional connectomes; (b) the two networks interact even in the absence of an explicit task; and (c) that the main sites of interactions between these two systems are the pSTS and the aIFG.

Our description of the connectivity fingerprint of the “normal” interplay between MS and AON is the key to comprehending the neural substrates underlying social cognition and the functioning of these networks. Resting‐state fMRI might easily be adopted to identify “abnormal” connectivity fingerprints in neuropsychiatric diseases—e.g., autism and schizophrenia—characterized by specific dysfunctional impairments in social cognition. This might provide a potential biomarker for identifying the specific “connectivity” fingerprints of different neuropsychiatric disease, linking behavioral impairment with neural function.

## Author Contributions

**Luciano Simone:** conceptualization, formal analysis, funding acquisition, supervision, validation, visualization, writing – original draft, writing – review and editing. **Enrica Pierotti:** formal analysis, writing – review and editing. **Eleonora Satta:** data curation, investigation, methodology, visualization, writing – review and editing. **Cristina Becchio:** writing – review and editing. **Luca Turella:** conceptualization, data curation, formal analysis, funding acquisition, investigation, methodology, project administration, supervision, validation, visualization, writing – original draft, writing – review and editing.

## Ethics Statement

Ethical approval for this study was obtained from the Ethics Committee of the University of Trento (Protocol number: 2018‐015) for research involving human participants. All participants were informed about the experimental procedures and provided written informed consent before experimentation.

## Conflicts of Interest

The authors declare no conflicts of interest.

### Peer Review

The peer review history for this article is available at https://www.webofscience.com/api/gateway/wos/peer‐review/10.1111/ejn.70082.

## Statistics and Reproducibility

All statistical procedures were performed by using open software. For each statistical step, the description of each parameter was provided.

## Supporting information


**Figure S1** Intra‐hemispheric rs functional connectivity of AON and MS functional ROIs from the HCP dataset.
**Figure S2** Inter‐hemispheric rs functional connectivity of AON and MS functional ROIs from the HCP dataset.

## Data Availability

The raw functional and structural data of this study may be made available upon request.
