# Supplementary material for: Resting‐State Functional Interactions Between the Action Observation Network and the Mentalizing System
Source: Eur J Neurosci. 2025 Mar 20;61(6):e70082. doi: 10.1111/ejn.70082 (PMC11926297; doi:10.1111/ejn.70082)
Supplement: Supplementary file 1 — Figure S1 Intra‐hemispheric rs functional connectivity of AON and MS functional ROIs from the HCP dataset. Figure S2 Inter‐hemispheric rs functional connectivity of AON and MS functional ROIs from the HCP dataset. [file EJN-61-0-s001.docx]

*Supplementary Materials for:*

***Resting-state functional interactions between the action observation network and the mentalizing system***

Luciano Simone^1^, Enrica Pierotti^2^, Eleonora Satta^2,3^, Cristina Becchio^4^, Luca Turella^2^

*^1 Department of Medicine and Surgery, University of Parma Via Voltuno 39, 43125 Parma, Italy;^*

*^2.Center for Mind/Brain Sciences (CIMeC), University of Trento, Corso Bettini 31, 38068 Rovereto (TN) Italy;^*

*^3. Laboratory for Autism and Neurodevelopmental Disorders, Center for Neuroscience and Cognitive Systems @UniTn, Istituto Italiano di Tecnologia, Rovereto;^*

*^4. Department of Neurology, University Medical Center Hamburg-Eppendorf, Martinistraße 52, 20246 Hamburg, Germany.^*

# Methods

## **Participants and MR data acquisition**

We downloaded the resting state fMRI and anatomical data of the Human Connectome Project dataset (HCP, D.C. Van Essen et al., 2012; David C. Van Essen et al., 2013) acquired on a 3 Tesla scanner. Of the original 1200 healthy participants, we considered only a subset 983 participants (avg age 28.8, std 3.7; 540 female). We selected the participants who were right-handed (Edinburgh handedness score higher than 0, (Oldfield, 1971) and performed one run of rs-fMRI (in Left-Right encoding phase). Each participant performed two runs of rs-fMRI acquired with different phase encoding, left-right (LR) and right-left (RL). We employed only the rs-fMRI runs with left-right encoding phase. Each run comprised 1200 volumes with a repetition time of 720 milliseconds (Smith et al., 2013). The total acquisition time of one run was 14.4 min. For a detailed description of the acquisition parameters of the fMRI dataset please refer to Van Essen et al. (2012).

## **Preprocessing and Functional connectivity estimation**

We adopted the minimally pre-processed HCP data (for detailed information see Glasser et al., 2013) which include a pre-processing pipeline including motion correction, coregistration to T1-weighted images and normalization to the MNI152 space. In addition, the dataset was further pre-processed by the application of the ICA-FIX algorithm: this analysis identifies specific components using independent component analysis (ICA); then, a classifier (FIX) trained on the HCP data was applied to remove artefactual components (Griffanti et al., 2014; Salimi-Khorshidi et al., 2014; Smith et al., 2013).

As in the original analysis, we wanted to describe the functional interplay within and between the mentalizing and action observation networks, so we extracted the timecourses of ROIs of the AON (vPM, dPM, pIFG, SPL, IPL, pSTS) and of the MS (TPJ, Prec, mPFC, ATL, aIFG). For this analysis, we created the ROIs adopting the MNI coordinates of the group analysis of the localizer (see Table 1) with radius of 8 mm. Then, we performed pairwise correlations (Person correlation coefficient) between the timecourse of these regions and normalized these correlation coefficients with the z Fisher transformation. As for the original analysis, we obtained a total of three connectivity matrices (11x11 ROIs) describing functional connectivity: within each hemisphere (left and right hemisphere separately) and across hemisphere. Then, we tested if the connectivity values at the group level were different from zero. To control for multiple comparisons across all possible ROIs connections within each connectivity matrix, we applied the False Discovery Rate approach (FDR-corrected p <0.05).The correlation between the connectivity matrices from the HCP and those from our study was calculated by the corr function in MATLAB.

**Results**

The results of the second level ROI-to-ROI analyses from the HCP dataset calculated by using group-level peak-activation coordinates from the Why/How task overlap with the functional networks identified in the subjects that actually performed the task. Indeed, within both hemispheres, the ROIs of each network (AON and MS) resulted functionally coupled mainly with ROIs of the same network. The pSTS and aIFG represent the only exceptions to this functional organization as they are functionally coupled also with MS and AON ROIs respectively (see Supplementary Figure 1).


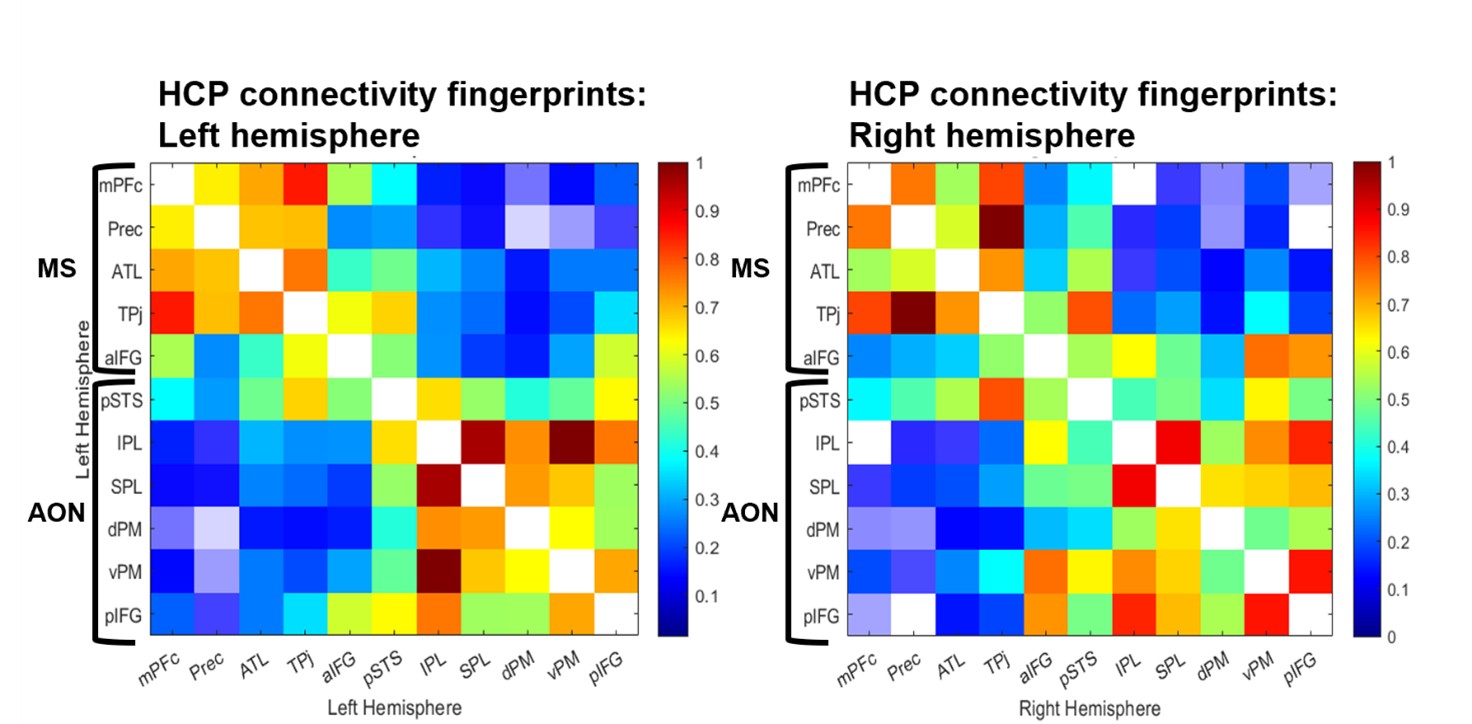


**Supplementary Figure 1. Intra-hemispheric rs functional connectivity of AON and MS functional ROIs from the HCP dataset.** The correlation matrices for the left and right ROIs of the MS and of the AON computed on the Human Connectome Project dataset. The ROIs correspond to the group-level coordinates of mentalizing and action-observation regions from our dataset. All significant correlations are reported in jet colormap whereas non-significant correlations are shown in white (FDR-corrected p-value <0.05). The diagonal (correlation of each ROI with itself) is represented in white. Color bar represents (Fisher z-score) Pearson correlations.

Similar results were obtained by computing inter-hemispheric functional connections namely by computing group-specific ROI-to-ROI analyses between regions located in different hemispheres. As shown in the Supplementary Figures 2, that depicts the functional connectome across hemispheres, although AON and MS ROIs mainly correlate with contralateral ROIs belonging to the same network, pSTS and aIFG showed hybrid coupling connecting with regions of both networks. To quantify the similarity between our ROI to ROI results and that obtained from the HCP dataset, we performed the correlation (corr matlab function) between the inter- and intra-hemispheric functional connectome from the two populations. The results show that both the intra-hemispheric (left: 0.8389, p<0,001; right: 0.7478, p<0,001) and interhemispheric (Across: 0.785, p<0,001;) functional connectivity from the two datasets were highly correlated.


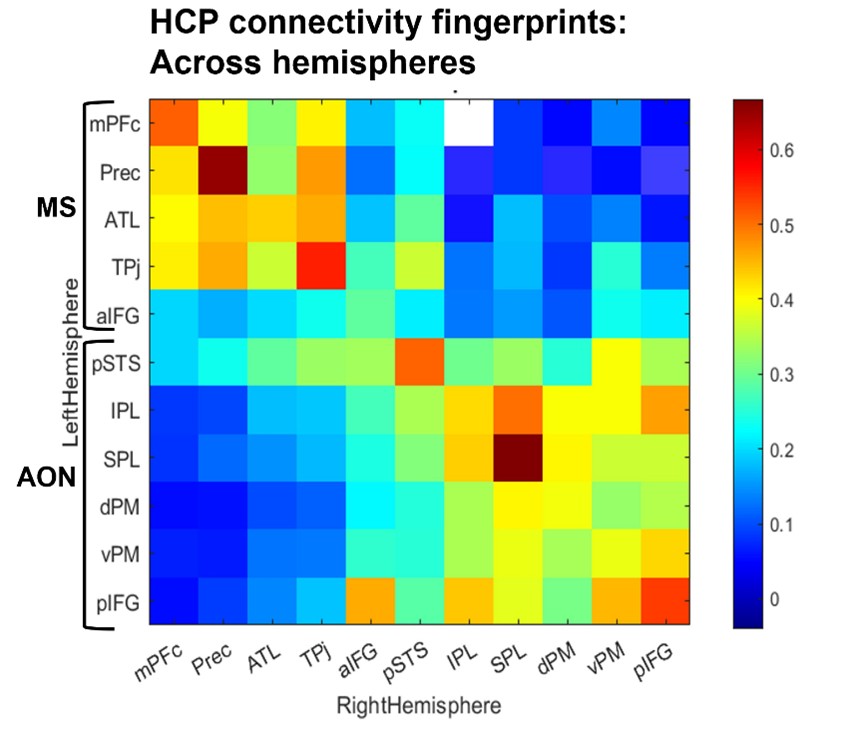


**Supplementary Figure 2. Inter-hemispheric rs functional connectivity of AON and MS functional ROIs from the HCP dataset.** The correlation matrix of the ROIs of the MS and of the AON from the Human Connectome Project dataset computed across hemispheres. The ROIs correspond to the group-level coordinates of mentalizing and action-observation regions from our dataset. All significant correlations are reported in solid color (jet colormap) whereas non-significant correlations are shown in white (FDR-corrected p-value <0.05). Color bar represents (Fisher z-score) Pearson correlations.

**References**

Glasser, M. F., Sotiropoulos, S. N., Wilson, J. A., Coalson, T. S., Fischl, B., Andersson, J. L., Xu, J., Jbabdi, S., Webster, M., Polimeni, J. R., Van Essen, D. C., & Jenkinson, M. (2013). The minimal preprocessing pipelines for the Human Connectome Project. *NeuroImage*, *80*, 105–124. https://doi.org/10.1016/j.neuroimage.2013.04.127

Griffanti, L., Salimi-Khorshidi, G., Beckmann, C. F., Auerbach, E. J., Douaud, G., Sexton, C. E., Zsoldos, E., Ebmeier, K. P., Filippini, N., Mackay, C. E., Moeller, S., Xu, J., Yacoub, E., Baselli, G., Ugurbil, K., Miller, K. L., & Smith, S. M. (2014). ICA-based artefact removal and accelerated fMRI acquisition for improved resting state network imaging. *NeuroImage*, *95*, 232–247. https://doi.org/10.1016/j.neuroimage.2014.03.034

Oldfield, R. C. (1971). The assessment and analysis of handedness: The Edinburgh inventory. *Neuropsychologia*, *9*(1), 97–113. https://doi.org/10.1016/0028-3932(71)90067-4

Salimi-Khorshidi, G., Douaud, G., Beckmann, C. F., Glasser, M. F., Griffanti, L., & Smith, S. M. (2014). Automatic denoising of functional MRI data: Combining independent component analysis and hierarchical fusion of classifiers. *NeuroImage*, *90*, 449–468. https://doi.org/10.1016/j.neuroimage.2013.11.046

Smith, S. M., Beckmann, C. F., Andersson, J., Auerbach, E. J., Bijsterbosch, J., Douaud, G., Duff, E., Feinberg, D. A., Griffanti, L., Harms, M. P., Kelly, M., Laumann, T., Miller, K. L., Moeller, S., Petersen, S., Power, J., Salimi-Khorshidi, G., Snyder, A. Z., Vu, A. T., … Glasser, M. F. (2013). Resting-state fMRI in the Human Connectome Project. *NeuroImage*, *80*, 144–168. https://doi.org/10.1016/j.neuroimage.2013.05.039

Van Essen, D.C., Ugurbil, K., Auerbach, E., Barch, D., Behrens, T. E. J., Bucholz, R., Chang, A., Chen, L., Corbetta, M., Curtiss, S. W., Della Penna, S., Feinberg, D., Glasser, M. F., Harel, N., Heath, A. C., Larson-Prior, L., Marcus, D., Michalareas, G., Moeller, S., … Yacoub, E. (2012). The Human Connectome Project: A data acquisition perspective. *NeuroImage*, *62*(4), 2222–2231. https://doi.org/10.1016/j.neuroimage.2012.02.018

Van Essen, David C., Smith, S. M., Barch, D. M., Behrens, T. E. J., Yacoub, E., & Ugurbil, K. (2013). The WU-Minn Human Connectome Project: An overview. *NeuroImage*, *80*, 62–79. https://doi.org/10.1016/j.neuroimage.2013.05.041
